# Anatomic Risk Factors for Patellofemoral Joint Instability: An Infographic as a Visual Learning Tool

**DOI:** 10.7759/cureus.53170

**Published:** 2024-01-29

**Authors:** Angelo V Vasiliadis, Theodore Troupis, Dimosthenis Chrysikos, Dimitrios Chytas, George Noussios

**Affiliations:** 1 Sports Trauma and Orthopaedic Department, St. Luke’s Hospital, Thessaloniki, GRC; 2 Department of Surgery, University of Athens Medical School, Athens, GRC; 3 Department of Anatomy, National and Kapodistrian University of Athens, Athens, GRC; 4 Department of Physiotherapy, University of Peloponnese, Sparta, GRC; 5 Department of Physical Education and Sports Science at Serres, Aristotle University of Thessaloniki, Thessaloniki, GRC

**Keywords:** trochlea dysplasia, patella alta, patella instability, patellofemoral, risk factors

## Abstract

Patellofemoral instability is a complex pathology with multiple risk factors, which affects mostly young females and may avert them from both activities of daily living and sports participation. Risk factors for instability include patella alta, trochlea dysplasia, abnormal lateral patellar tilt, and increased tibial tuberosity-trochlea groove distance. The knowledge of these anatomical abnormalities is the key to identifying the problem and succeeding in treating the patients.

## Editorial

Patellar instability is a potentially debilitating condition that frequently affects female adolescents and negatively influences both their activities of daily living and sports participation [1]. It is characterized by anterior knee pain, recurrent subluxation episodes, and a subjective feeling of locking or catching [1,2]. A thorough physical examination is crucial and includes medical history and functional tests, to assess possible malalignment, joint laxity, and range of motion [2]. Symptoms can be reproduced during the patellar apprehension test and it is positive when there is pain and reflex contraction of the quadriceps muscle to prevent the lateral subluxation of the patella [1,3]. Management of patellar instability is complex and begins with the identification of risk factors [1,2]. Multiple risk factors have been identified for patellar instability, while these can be classified into major (which in the majority of the cases are osseous) and secondary (including soft tissue envelope) risk factors [1,4]. Among them, patella alta, trochlea dysplasia, excessive lateral patellar tilt, and increased tibial tuberosity-trochlea groove (TT-TG) distance have been proposed as the main risk factors for patellofemoral instability [4].

Firstly, the height of the patella can be assessed with the knee in 30 degrees of flexion, either on lateral radiograph, sagittal computed tomography (CT), or magnetic resonance imaging (MRI) [4,5] Multiples ratios can be calculated and provide information about the height of the patella [5]. However, the Caton-Deschamps index is the preferred one due to its advantages in quantifying patella height changes after tibial tubercle osteotomy, allowing patella height measurements for various degrees of knee flexion, different knee sizes, variable skeletal maturation, and patellar pole abnormalities [4]. A ratio of the Caton-Deschamps index above 1.3 is considered indicative of patella alta [4]. Secondly, trochlea dysplasia has been identified as the main anatomical risk factor with the strongest association with patellar instability and is defined as a shallow or flattened groove with decreased resistance to lateral patellar translation [1]. Trochlea dysplasia can be assessed on CT or MRI, whereas, the thresholds for identifying trochlear dysplasia are established based on the sulcus angle > 145° [6,7]. Moreover, patella tilt is defined as the angle formed between the plane of the posterior femoral condyles and a line drawn connecting the medial and lateral borders of the patella [7]. According to the literature, a value of more than 20 degrees as a threshold of abnormal lateral patella tilt is suggested [1,7]. Finally, elevated TT-TG distance can negatively impact the biomechanics of the patellofemoral joint and predispose to patellar instability [8]. Increased TT-TG distance of more than 20 mm, evaluated on CT scan, is reportedly associated with pathologic patellofemoral instability [1,8].

Besides osseous support, the stability of the patella is also affected by the soft tissue envelope. Hypoplastic or absent vastus medialis oblique (VMO) muscle, overpull of lateral structures, such as iliotibial band (ITB) and vastus lateralis (VL), and insufficient medial patellofemoral ligament (MPFL) can lead to a chronic patellar tilting and contribute to a tight and thick lateral retinaculum and further patellar instability [1,4]. The VMO muscle seems to be an important dynamic stabilizer for the patella and plays a crucial role in balancing the forces from the lateral structures, such as ITB and VL [1,9]. Muscle atrophy of the VMO leads to excessive lateral maltracking of the patella, resulting in primary patella dislocation [4,9]. Additionally, MPFL has an important role in minimizing the total forces against lateral patella displacement and it is considered to be the most important anatomical structure in the medial region of the patellofemoral joint [9]. Chronic insufficient MPFL can result in an increased risk for future patellar instability and recurrence of patellar dislocation [1,7].

**Figure 1:**
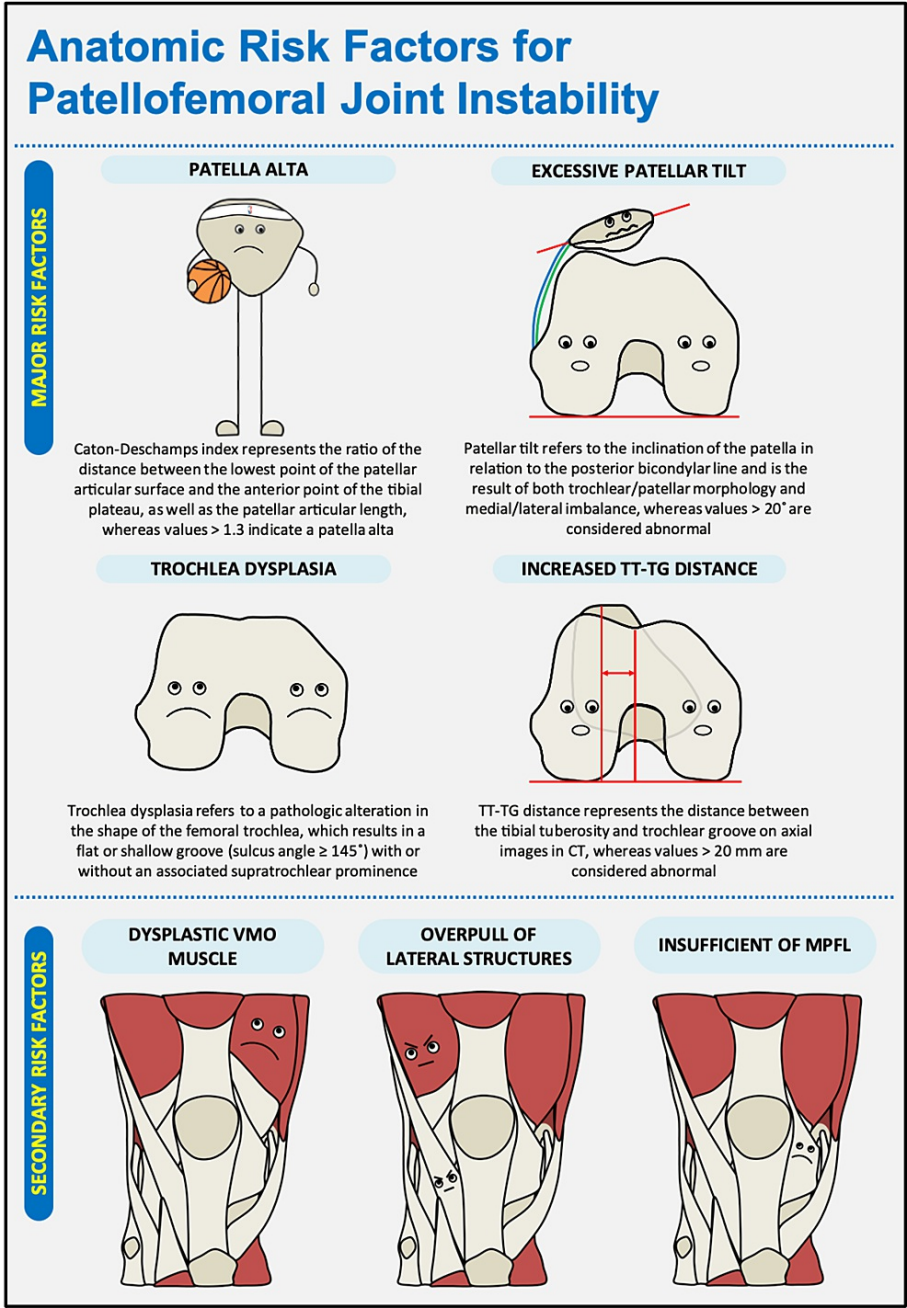
Anatomic risk factors for patellofemoral joint instability. Abbreviations: TT-TG, tibial tuberosity to trochlea groove; VMO, vastus medialis oblique; MPFL, medial patellofemoral ligament; CT, computed tomography. The figure is created by the first author.

Patellar instability is a common condition in children and adolescents, ranging from mild discomfort and mal-tracking to lateral patellar dislocation. A number of anatomical risk factors have been described and investigated in the literature, consisting of osseous and soft tissue anatomical risk factors (Figure 1). It is important to note that better results can be achieved when improving the management of patellofemoral joints by addressing all aspects of the instability. Deep knowledge of anatomic variations and abnormalities of the patellofemoral joint, which may predispose to patellar instability, is crucial to identifying the problem and choosing the appropriate treatment for each patient.

## References

[1] Dejour DH, Mesnard G, Giovannetti de Sanctis E (2021). Updated treatment guidelines for patellar instability: "un menu à la carte". J Exp Orthop.

[2] Kerzner B, Gursoy S, Dasari SP, Fortier LM, Yanke AB, Chahla J (2022). Trochlear osteochondral shell allograft technique to treat trochlear dysplasia in the setting of chondral damage and chronic patellar instability. Arthrosc Tech.

[3] Hiemstra LA, Kerslake S, Lafave MR (2021). Patellar apprehension is reduced in most but not all patients after successful patellar stabilization. Am J Sports Med.

[4] Dietrich TJ, Fucentese SF, Pfirrmann CW (2016). Imaging of individual anatomical risk factors for patellar instability. Semin Musculoskelet Radiol.

[5] Kwak YH, Park SS, Huser AJ (2022). Evaluation of age group and sex differences in the measurement of patellar height of pediatric knee in a Korean population. Front Pediatr.

[6] Batailler C, Neyret P (2018). Trochlear dysplasia: imaging and treatment options. EFORT Open Rev.

[7] Askenberger M, Janarv PM, Finnbogason T, Arendt EA (2017). Morphology and anatomic patellar instability risk factors in first-time traumatic lateral patellar dislocations: a prospective magnetic resonance imaging study in skeletally immature children. Am J Sports Med.

[8] Brutico J, Paul RW, Wright M (2023). Preoperative patella alta on Caton-Deschamps index is a predictor of outcome following isolated medial patellofemoral ligament reconstruction. Arthrosc Sports Med Rehabil.

[9] Shu L, Yang X, He H, Chen B, Chen L, Ni Q (2021). Morphological study of the vastus medialis oblique in recurrent patellar dislocation based on magnetic resonance images. BMC Med Imaging.

